# Parasitic egg recognition using convolution and attention network

**DOI:** 10.1038/s41598-023-41711-3

**Published:** 2023-09-02

**Authors:** Nouar AlDahoul, Hezerul Abdul Karim, Mhd Adel Momo, Francesca Isabelle F. Escobar, Vina Alyzza Magallanes, Myles Joshua Toledo Tan

**Affiliations:** 1 Computer Science, New York University, Abu Dhabi, United Arab Emirates; 2 Faculty of Engineering, Multimedia University, Cyberjaya, Malaysia; 3 Fleet Management Systems and Technologies, Istanbul, Turkey; 4 Department of Natural Sciences, University of St. La Salle, Bacolod, Philippines

**Keywords:** Machine learning, Medical imaging

## Abstract

Intestinal parasitic infections (IPIs) caused by protozoan and helminth parasites are among the most common infections in humans in low-and-middle-income countries. IPIs affect not only the health status of a country, but also the economic sector. Over the last decade, pattern recognition and image processing techniques have been developed to automatically identify parasitic eggs in microscopic images. Existing identification techniques are still suffering from diagnosis errors and low sensitivity. Therefore, more accurate and faster solution is still required to recognize parasitic eggs and classify them into several categories. A novel Chula-ParasiteEgg dataset including 11,000 microscopic images proposed in ICIP2022 was utilized to train various methods such as convolutional neural network (CNN) based models and convolution and attention (CoAtNet) based models. The experiments conducted show high recognition performance of the proposed CoAtNet that was tuned with microscopic images of parasitic eggs. The CoAtNet produced an average accuracy of 93%, and an average F1 score of 93%. The finding opens door to integrate the proposed solution in automated parasitological diagnosis.

## Introduction

Parasitic infections are among the main public health problems worldwide, especially in tropical and subtropical countries^1,2^. According to the World Health Organization’s Global Health Estimates in 2020, infectious and parasitic diseases are among Africa's leading causes of death^3^. There are three main classes of parasites that can cause disease in humans: protozoa, helminths, and ectoparasites^2^. Helminth infection has threatened over 800 million individuals throughout the world (CDC). Humans in developing countries, such as Sub-Saharan Africa, South America, and East Asia, are infected with medically significant nematodes, trematodes, and cestodes^3^. While these diseases manifest in a broad range of clinical manifestations, the elimination and early classification for the prevention of pathogenic helminths offers significant socio-economic benefits.

Microscopy is one of the most commonly used conventional methods in classifying and diagnosing parasitic diseases and is exclusively dependent on medical technicians for examinations^4,5^. However, microscopy-based parasite identification and quantification pose a lot of issues^5^. It is challenging, time-consuming, labor-intensive, error-prone and requires well-trained researchers for identification^5–7^. Because of the variances and ambiguities in the parasites' shape, density, and staining color, human experts find parasitic examination through microscopic photographs difficult^2,8^. Furthermore, these conventional methods lack a data-sharing framework and historical records of diagnosis. Consequently, the development of an automated diagnostic system would be a major leap in assisting traditional diagnosis.

With technological advancements and the development of image processing techniques and computer vision, artificial intelligence has been successfully used in various applications, such as face recognition, natural language processing, and biomedical image analysis^2,9–11^. Resulting in automated diagnosis systems becoming more attainable. Many studies have implemented systems to analyze the micrographs of the samples based on machine learning, e.g. support vector machine (SVM)^12^ and artificial neural networks (ANN)^12,13^. These traditional machine learning methods do not need complex structures, but they rely largely on selectively selected information. For this reason, fine-tuning the features in the feature extraction step will take a lot of time and effort. On the other hand, deep learning-based algorithms have been increasingly popular in the previous decade as computer performance and the number of available image datasets have both improved^14,15^. Deep learning has demonstrated remarkable efficiency in a variety of disciplines, including text recognition, computer-assisted diagnosis, facial identification, and drug development^16^. Deep learning, particularly the Convolutional Neural Network (CNN), stimulates novel parasite classification research in the parasite egg detection task because of its promising performance and speed in object recognition^4,5,8,13,17,18^. By learning important features automatically from a vast amount of data that represents the desired behavior of the data, CNNs offer an advantage over conventional feature extraction^18^. CNNs have exhibited great accuracy in a variety of pathogen identification applications, including malaria, tuberculosis, and intestinal parasite detection^19^. Furthermore, methods such as Single Shot MultiBox Detector (SSD), U-Net, and Faster Region-based Convolutional Neural Network (Faster-CNN), which are methods based on object detection and segmentation also flourished^4,8^. These superior deep learning approaches encouraged us to build a platform for identifying and quantifying helminth eggs that were faster and more automated.

## Previous works

Machine learning is a field of artificial intelligence built upon the theory of statistics to provide predictive or descriptive information on given data^20^. It is able to execute such tasks by making inferences from input samples or data without necessarily incorporating specific instructions into the algorithm. Applications of machine learning for microscopic analysis in parasitic identification have exhibited promising results for the fields. Conventional methods involve image processing techniques for data extraction followed by analysis using machine learning.

To detect parasite eggs, Bruun et al.^21^ employed elliptic filters. These filters were created based on the average size of parasite eggs and hand-selected rotation angles. These handcrafted traits had a classification accuracy of over 93%. Delas Penas et al.^17^ explored the use of a convolutional neural network framework, You Only Look Once (YOLO) in detecting three helminth eggs of *Schistosomiasis, Trichuriasis*, and *Ascariasis* in stool samples. Avci and Varol^22^ first proposed MC-SVM as a new method for classification of human parasitic eggs using micrographs. MC-SVM is composed of four stages. These are the pre-processing stage, feature extraction stage, classification stage, and testing stage^22^. Yang et al.^23^ uses an ANN in helminth egg classification, achieving 90.3% validation accuracy. Preprocessing involved median filtering, binary thresholding, segmentation, and feature (size, shape, eggshell) extraction. While^12^, in a recent study, classified *Ascaris* eggs using ANN coupled with Multi-class Support Vector Machine (MC-SVM). The experimental findings indicated a 95% and 93% accuracy rate in identifying *Ascaris* eggs, respectively. Additionally, on a much recent study, Ray et al.^24^ explored different types of features examined using three different classifiers viz SVM, ANN and k-nearest neighbors (kNN), and utilized micrographs of three different types of parasitic eggs namely *Ascaris Lumbricoides*, *Necator Americanus* and *Trichuris Trichiura*. SVM using texture and shape-based features achieves the highest classification accuracy of 96.5%. Logistic regression was also used for multiclass parasitic recognition^25^; in the study they used geometric and brightness features extracted through an extensive sequence of image processing methods. Overall, the proposed process attained a specificity of 98%. For identification and classification of microscopic nematode eggs, Akintayo et al.^26^ used a deep convolutional selective Autoencoder architecture. According to their findings, the Autoencoder can detect 92% of less-clustered images and 96% of high-clustered images. They did note, however, that this method has a high computational cost that can be minimized by employing a more powerful computer.

In intestinal parasite detection and classification^1^, distance regularized level set evolution (DRLSE) and circular hough transform (CHT) were selected for object recognition and segmentation. Histogram oriented gradient (HOG) was then applied for feature extraction. The proposed classifier was a combination of fuzzy classification techniques and artificial neural networks, its accuracy measuring 100%.

Deep learning is a recent advancement in machine learning that surpasses the limitations found in traditional methods. It is a neural network composed of multiple processing layers capable of gathering complex representations of raw data^27^. This eliminates the need for tedious preprocessing techniques for feature extraction and demonstrates high exceptional performance with abundant data. In a comparative study by^6^, an image processing-SVM model and VGG-16, a pre-trained deep neural network, were used for parasite classification wherein VGG-16 outperforms SVM. Currently, convolutional neural networks (CNN) are most frequently used in vision tasks. Butploy et al.^13^ proposed a 3-layer CNN for *A. lumbricoides*. egg classification, measuring 93% in accuracy involving three classes. Since deep learning requires extensive data for training, challenges in limited datasets can be solved through transfer learning. This takes a neural network pre-trained on a large dataset and applies the architecture and learned weight on a new, but similar task. Examples of transfer learning for parasite classification include VGG-16^6^, GoogleNet, AlexNet, and Resnet50^5,28^.

Akintayo et al.^26^ designed a novel end-to-end Convolutional Selective Autoencoder (CSAE) to identify a parasitic worm, soybean cyst nematode (SCN). CSAE has two parts—the encoder and the decoder. CSAE architecture was developed for this rare-object detection class of problems. The ‘selectivity’ feature of CSAE possess remarkable detection speed, and accuracy in identifying SCN eggs across a wide range of samples e.g., soil, debris as it is trained to only reconstruct an 'egg' pattern while masking/rejecting all other patterns in the input image. Simon et al.^29^ introduced a new architecture consisting of a shallow CNN appended with a single recurrent layer. Results showed better performance in the proposed models than in the state-of-the-art models. Wiem et al.^18^ proposed a fusion decision method to identify parasitic eggs and utilized CNN by feeding the outputs for the discrete transform as input.

Suwannaphong et al.^5^ presented a CNN-based transfer learning model to improve the effectiveness of automated parasite classification in low-resolution. Two networks, AlexNet and ResNet50 were examined and a patch-based technique with a sliding window was utilized to search for the egg location. Huo et al.^30^ proposed an automatic recognition algorithm based on YOLO for parasitic eggs and achieving an average accuracy of 99.4%. On the other hand, Górriz et al.^31^ trained a U-net model for the classification of *leishmania* parasites into promastigotes, amastigotes and adhered parasites. Najgebauer et al.^32^ proposed a technique that uses a fully convolutional network (FCN) to analyze the complete sample space and give a class to each pixel in the image. The program was taught to identify parasite eggs and differentiate them from the adjacent or overlapping pollution. Roder et al.^7^ implemented Deep Belief networks (DBN) for automatic classification of intestinal parasites viz eggs, larvae and protozoa. Considering the impurities of the fecal samples and unbalanced classes used, the datasets nevertheless, garnered promising results. Lastly, Nkamgang et al.^1^ trained a neuro-fuzzy classifier according to a speeded up scaled conjugate gradient algorithm for recognition and classification of twenty human intestinal parasites. Achieving a recognition rate of 100%.

The objective of this paper is to propose a solution to classify parasitic eggs. This solution should meet specific requirements including ability to recognize various categories, high classification accuracy, low inference time, and memory efficiency. Table l demonstrates various methods used in the literature for parasitic egg classification. The existing methods showed several drawbacks such as complex structure, limitation of number of categories, high training time, low accuracy, computational cost and time, and uninformative features. In this work, we found that CoAtNet0 was able to address these drawbacks for parasitic egg classification task with simpler structure, higher accuracy, lower computational cost and time, better informative features, and capability to recognize various eggs categories.

**Table 1:** Various classification methods with their drawbacks and advantages.

Method	Pros	Cons
Traditional human examination^5–7^	Accurate with human experts	*Challenging *Time-consuming, * labor-intensive *Error-prone *The need of well-trained researchers
Machine learning: support vector machine^12^ and artificial neural networks^12,13^	These methods do not need complex structures	Fine-tuning the features will take a lot of time and effort
Deep learning-based algorithms^14,15^ particularly CNN, Single Shot MultiBox Detector (SSD), U-Net, and Faster Region-based Convolutional Neural Network^4,8^	*Promising performance and speed in recognition *Learning important features automatically *CNNs offer an advantage over conventional feature extraction	*It needs powerful computer *Number of available image datasets is sensitive
CNN You Only Look Once (YOLO)^17^	Good detection and classification accuracy	Recognizing only three categories
A deep convolutional selective Autoencoder architecture^26^	Identification and classification of microscopic nematode eggs	A high computational cost
CNN-based transfer learning model Two networks, AlexNet and ResNet50 were examined and a patch-based technique with a sliding window was utilized to search for the egg location^5^	Improve the effectiveness of automated parasite classification in low-resolution	More time required to slide the window across high resolution images
EfficientDet with EfficientNet-v2 backbone^33^	High accuracy with localization capability	Complex architecture of two backbones used to extract features and fuse the decision produced in the output layers
EfficientNet-B7 with layers frozen and support vector machine (SVM) tuned^33^	Less training time because no layers finetuning	Trained with dataset that is not related to task studied

This paper highlights an interesting challenge for the research community. It contributes to the body of knowledge as follows:

1. Various convolutional neural networks such as DenseNet121^34^ and EfficientNet-B4^35^ were explored. These pre-trained CNNs were fine-tuned with parasitic eggs dataset for classification purposes.
2. Vision Transformer^36^ which uses self-attention mechanism was demonstrated. It was fine-tuned with parasitic eggs dataset for classification purposes.
3. A CoAtNet (Convolution and Attention Network) was proposed^37^ for parasitic egg recognition in microscopic images for classifying parasitic eggs. The pre-trained CoAtNet was fine-tuned with parasitic eggs dataset for classification purposes.
4. This work presents a novel attempt to utilize the concept of combining convolution and attention in medical applications such as parasitic egg recognition.
5. This work targets a novel dataset that was proposed in ICIP2022 challenge to recognize eleven types of parasitic eggs acquired under various complex conditions such as illuminations and resolutions.

This paper is organized as follows: “Materials and Methods” describes the parasitic eggs dataset. Additionally, it discusses numerous convolutional neural networks and self-attention vision transformer. Additionally, we demonstrated convolution and attention network. In “Experiments and results”, the experiments conducted are described to analyze results in detail. We performed an evaluation and comparison between various deep learning models. Finally, "Conclusion and future work". summarizes the work presented in this paper by giving readers a glimpse into potential improvements in the future.

## Materials and methods

This section demonstrates the dataset used in this work for parasitic egg classification. Additionally, it describes various CNN based models utilized as baseline methods. Furthermore, it explores the proposed solution of convolution and attention network to be compared with the baselines.

### Dataset overview

**Figure 1:**
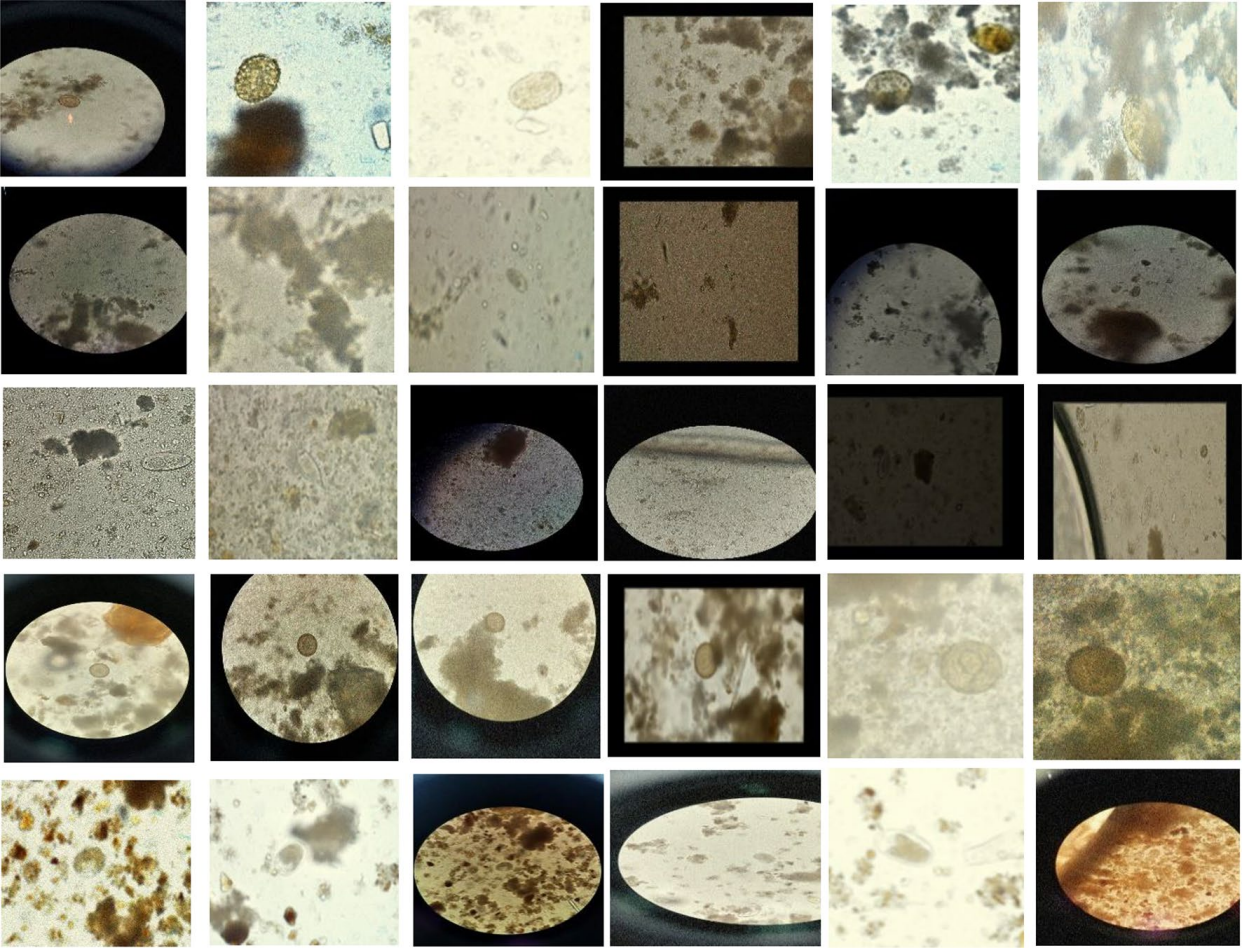
several samples of microscopic images including various condition such as illuminations, resolutions, sizes of eggs, and blurring^38–40^.

A completely unique dataset called Chula-ParasiteEgg-11 was proposed for an ICIP 2022 competition^38–40^. The dataset includes 11 categories of different types of parasite eggs from faecal smears, with an average diameter between 15 and 100 μm, all labeled in bounding boxes. The following are among the categories: *A. lumbricoides*, *Capillaria philippinensis*, *Enterobius vermicularis*, *Fasciolopsis buski*, *Hookworm egg*, *Hymenolepis diminuta*, *H. nana*, *Opisthorchis viverrine*, *Paragonimus* spp., *Taenia* spp. egg, and *T. trichiura*^38–40^. With 1000 micrographs for the training set and 250 micrographs for the testing set for each category, the dataset is the largest collection of its kind. Multiple devices such as a Canon EOS 70D camera body with Olympus BX53 microscopes, a DS-Fi2 Nikon camera body with Nikon Eclipse Ni microscopes, Samsung Galaxy J7 Prime phone, and iPhone 12 and 13 with either 10× eyepiece lenses of Nikon Eclipse Ni or Olympus BX53 devices were used to collect the micrographs of the samples. As a result, the resolution, illumination, and setting conditions of each image vary. And this variation could render the detection more reliable^38–40^. Figure 1 shows several samples. Table 2 shows the number of samples for each category of parasitic eggs.

**Table 2:** Chula-ParasiteEgg-11 dataset class distribution.

	Number of samples
*Ascaris lumbricoides*	200
*Capillaria philippinensis*	200
*Enterobius vermicularis*	200
*Fasciolopsis buski*	200
*Hookworm egg*	200
*Hymenolepis diminuta*	200
*Hymenolepis nana*	200
*Opisthorchis viverrine*	200
*Paragonimus *spp.	200
*Taenia *spp. egg	200
*Trichuris trichiura*	200
Total	2200

Parasite eggs vary between 20 and 80 μm dimensions, and they are usually seen under microscopes only. To detect eggs in microscopic images, long time of visual analyses is required by expert human. This task is very prone to human errors. Several characteristics used to identify parasite eggs including size, shape, shell thickness, surface structure and the presence of an operculum and polar plugs as shown in Fig. 1. The proposed solution of using CoAtNet0 was able to extract features from various eggs by extracting features of shape, shell thickness, surface structure, and operculum and polar plugs automatically without need of expert knowledge. The capability of model to extract these features outperform the human expert capability which is usually prone to mistakes.

### Methods

**Figure 2:**
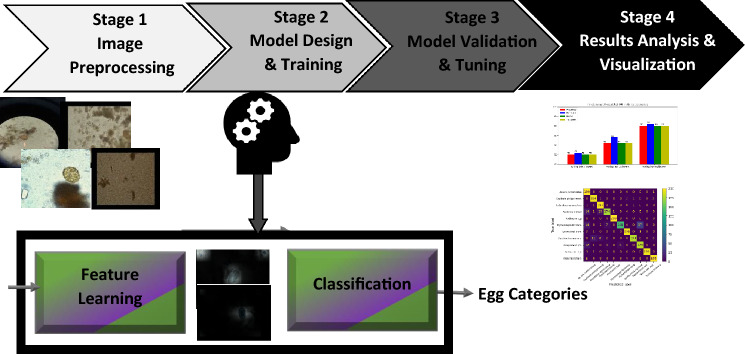
The pipeline of egg classification task.

This section discusses the methodology of this work including the stages required to learn the mapping between the images of parasitic eggs and their corresponding categories as shown in Fig. 2.

Convolutional neural networks (CNNs) have shown superior performance with high generalization compared to previous hand-crafted features methods. Therefore, they were selected in the first set of experiments to train and evaluate them with parasitic egg dataset. After conducting experiments related to state-of-the art CNNs such as EfficientNet and DenseNet with various numbers of layers trained, we carried out another set of experiment to explore vision transformer as self-attention mechanism to study the potential improvement that this high capacity model may produce. Finally, we moved to demonstrate the superior performance of CoAtNet in various applications conveys behind the combination of two techniques including attention and convolution in one model. Therefore, we targeted to benefit from this combination in parasitic egg classification task. The details of each method are described in the following sections.

In this work, we considered real world implementation of the proposed methods to reduce the computation cost which has impact on the inference speed. There are numerous versions of EfficientNet such as EfficientNetB0, EfficientNetB4, and EfficientNetB7. In this study, EficieneNetB4 was selected because it has 19 million parameters compared to 5.3 million in EfficientNetB0 and 66 million in EfficientNetB7. In other words, EfficientNetB4 can balance between good performance and high speed. As known, fewer parameters leads to faster inference and more parameters lead to higher classification accuracy. Additionally, DenseNet121 was selected among other versions of DenseNet because it has 7 million parameters which can also balance between good performance and high speed. To move to self-attention network, vision transformer^36^ base version with 16 × 16 patch size was selected due to lower parameters compared to large version. Furthermore, CoAtNet0 was chosen because it has 25 million parameters which is smaller than other CoAtNet versions.

### EfficientNet CNN

EfficientNet is a family of convolutional neural networks (CNN) which were built on using a newer approach for scaling up models, particularly designed for image recognition. Previous methods involve adding more layers or through image resolution. Scaling up one of the following: depth, width, or image size is a common practice. Tan and Le^35^ introduced a method that scales all three in a uniform fashion following a fixed proportion. This was able to resolve several limitations found in older models and improve in accuracy on ImageNet^41^. EfficientNet consists of models B0–B7, starting with the baseline (B0) with its succeeding scaled up versions. In scaling dimensions, each feature held the ability to improve model accuracy but was met with restraints. It is presumed that more depth, or more layers would increase performance. However, this would require more training data^42^ and computational power. Additionally, this method is not the only means of improving CNNs; Ba and Caruna^43^ find that it is possible to train shallow neural networks to execute similarly to elaborate deeper CNNs. In a test assessing the scalability of each dimension, authors^35^ observed that increase in width resulted in problems with analysing higher level features while in using very high resolutions, accuracy gain begins to stagnate. Overall, experimental results indicate that accuracy gain declines as models continue to expand when scaling only one dimension of the three.

In creating an improved model, it was important to ensure that all dimensions were balanced when scaling CNN. This was made possible by a compound scaling method wherein the compound coefficient φ is used to uniformly scale the dimensions as determined by the user. In classifying ImageNet, EfficientNet achieved accuracy scores equivalent to other state-of-the-art models such as ResNet, Inception-v3, and DenseNet while using much fewer parameters and having reduced FLOPs (floating point operations). In contrast to other CNNs, EfficientNets also run much faster. When used for transfer learning on other datasets including CIFAR-100, Food-101, etc. EfficientNets also exhibited remarkable results.

Due its success on ImageNet and other extensive datasets, EfficientNet has been used over other applications in various domains. In medical imaging, EfficientNets were found to be superior in several classification of magnetic resonance imaging and X-ray imaging tasks. In chest abnormality identification featuring pneumonia, COVID-19, and normal lungs EfficientNetB0 performed higher than VGG16 and InceptionV3^44^. For MRI brain tumor recognition fine-tuned EfficientNetB0 also achieved the highest in performance^45^. In another study, EfficientNetB2 was found to be most suitable for breast cancer histopathology classification against other EfficientNets^46^. Ensemble methods also benefit EfficientNets; in a study for malaria diagnosis, infected cell micrographs were successfully classified^47^. These findings along with transfer learning results on large benchmark datasets support the potential of using EfficientNets for more computer vision tasks.

### DenseNet CNN

In the last few decades, deep CNN architecture has achieved many breakthroughs in image classification tasks^48,49^. However, as the CNN gets deeper, when the input information goes through many layers, there is a possibility to ‘wash out’ or vanish by the time it reaches the end (or the beginning) of the network. While various designs have varied network topologies and training methods, they still have one thing in common: they consistently make shorter pathways from earlier layers to later layers. Accordingly, Huang et al.^34^ developed a new CNN architecture referred to as Dense Convolutional Network (DenseNet) which aims to increase the depth of deep learning networks while also improving training efficiency by using shorter connections between the layers.

In DenseNet architecture, each layer is connected to every other layer to ensure maximum information flow between the layers of the network^34^. Each layer receives extra inputs from all earlier layers and transmits its own feature-maps to all later layers in order to maintain the feed-forward structure of the architecture. Contrary to Resnets, DenseNet concatenates the features rather than combining them through summation^50^. Thus, instead of just the L, in traditional architectures, there are L(L+1)/2 direct connections in DenseNet. To successfully facilitate both down-sampling in the architecture and feature concatenation, the size of the feature maps should be uniform. This was made possible by dividing the network into multiple densely connected dense blocks where feature map size remains the same. Now, transition layers, the layers between blocks can perform the convolution and pooling operations outside the dense blocks, while inside the dense block is able to perform feature concatenation. As opposed to current network topologies, DenseNet may have very narrow layers, for example, k = 12. Where network growth rate is referred to as the hyper parameter k. As each layer produces concatenated k feature maps, the number of inputs is quite high and has huge computational requirements. To increase the efficacy, DenseNet utilizes Bottleneck layers. Four DenseBlocks with variable numbers of layers make up each architecture. For instance, while DenseNet-169 has (6, 12, 32, 32) layers, DenseNet-121 has (6, 12, 24, 16) layers in its four dense blocks. A Classification Layer follows the fourth dense block, accepting the feature maps from all network layers to carry out the classification. The ultimate goal of DenseNet is to utilize features from every layer to improve model performance and robustness using a standard dataset while requiring minimal computational labor and a much lower model size. However, it should also be noted that the increasingly massive number of layers can result in explosive growth during training^51^.

Due to its dense connections between layers, and improved model performance, DenseNet has been preferred over any applications in a variety of fields. In medical image classification, DenseNet is the first one to successfully perform anatomical segmentation of the whole brain using MR images^52^. In metastatic cancer image classification, DenseNet achieved superior performance over the state-of-the-art approaches^53^.

In another study, DenseNet was used in predicting COVID-19 patients from CT images^54^. In another study, DenseNet was used for classification of COVID-19 cases in medical imaging^55^. Furthermore, a sparsely connected DenseNet was used for malaria parasite detection^56^.

### Vision transformer

A vision transformer is a deep learning model that is used for image classification and was inspired by Dosovitskiy et al.^36^. In this paper, a parasitic egg image that has a sequence of patches encoded as a set of words was applied to the transformer encoder. The original image’s patches N = (H × W)/P^2^ were extracted with a fixed patch size (P, P) where P = 16, W is the image width, H is the image height, and N is the number of patches. The extracted patches were flattened and each patch x_p_ belonged to R^P2.C^, where C is the number of channels. As a result, the 2D image was converted into a sequence of patches x∈R^N×(P2.C)^. Each patch in the sequence x was mapped to a latent vector with hidden size D = 768. The architecture of transformer’s encoder with L blocks, each block containing alternating layers of multi-head self-attention (MSA) and multi-layer perceptron (MLP) blocks. The layer normalization (LN) was applied before every block, while residual connections were applied after every block.

#### Convolution and attention

State-of-the-art (SOTA) Convolutional Neural Networks (ConvNets) (e.g., ResNet50^57^) were the dominating deep architecture for performing computer vision tasks (e.g., object detection, semantic segmentation, image classification) due to their properties of proper inductive bias, efficiency, and generalization. However, Vision Transformer (ViT) and its variants^58^ were explored in the benchmark dataset ImageNet-1k^41^ and yielded promising results, but the performance of ViT still falls behind ConvNets due to its property of higher capacity at scale than ConvNets, thus it needs a very large-scale dataset in order to achieve comparable results to SOTA ConvNets. Recently, CoAtNet^37^ was explored in ImageNet and it outperformed ViT and its variants. Additionally, its performance nearly matched the SOTA ConvNets (e.g., EfficientNet-V2^59^) on ImageNet-1k only. CoAtNet combines the properties of ConvNets (e.g., inductive bias, generalization, efficiency) with the properties of Vision Transformers (e.g., capacity). CoAtNet was found to achieve high performances across different data sizes, and specifically, under the low-data system. CoAtNet inherited the generalization capability from Convolutional networks and superior scalability from transformer models. It was able to match huge version of Vision transformer-pre-trained on 23 × larger dataset. Given enough data, CoAtNet achieved faster convergence and improved efficiency with less computation to outperform Vision transformer.

Several techniques were embedded in CoAtNet including self-attention and transformers which have been widely adopted for neural language processing and recently for different vision tasks. Additionally, relative attention (rel-attenttion) in CoAtNet belongs to the input-independent version which is computationally cheaper than the input-dependent version. The idea of combining convolution and self-attention for vision recognition usually enhances the accuracy with extra computational cost. On the other hand, CoAtNet with relative attention is a mixture of depthwise convolution and content-based attention combined into a single computation unit with less cost^37^.

MBConv block employs depthwise convolution^37^ to capture the spatial interaction because it can be effectively merged into attention layers with simple relative attention. convolution depends on a fixed kernel to collect information from a local receptive field y.

1 $${y}_{i}=\sum_{j\in L (i) }{w}_{i-j \odot }{x}_{j} (\mathrm{depthwise\, convolution})$$

where x_i_, y_i_ ∈ R^D^ are the input and output at position i respectively, and L(i) denotes a local neighborhood of i, e.g., a 3 × 3 grid centered at i in image processing.

Self-attention allows the receptive field to be the entire spatial locations and computes the weights based on the re-normalized pairwise similarity between the pair (x_i_, x_j_)^37^:

2 $${y}_{i}=\sum_{j\in g }\frac{\mathrm{exp}({x}_{i}^{T} {x}_{j})}{\sum_{k\in g}exp ({x}_{i}^{T} {x}_{k})} {x}_{j} (\mathrm{self\, attention})$$

where g indicates the global spatial space.

When the multi-stage layout is used, we mimic ConvNets to construct a network of 5 stages (S0, S1, S2, S3 and S4). The first stage S0 is a simple 2-layer convolutional Stem and S1 employs MBConv blocks with squeeze-excitation due to large spatial size. Starting from S2 through S4, we consider either the MBConv or the Transformer block, but the convolution stages must appear before transformer stages to process the local patterns.

**Figure 3:**
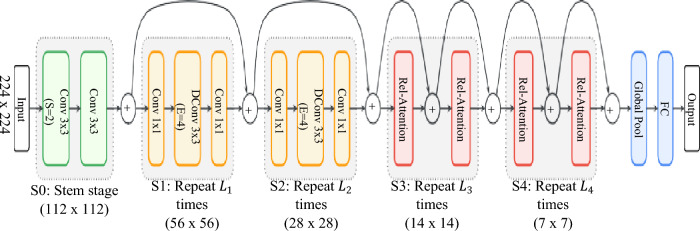
CoAtNet architecture^37^.

Considering generalization, model capacity, transferability and efficiency, simply stacking convolutional and attention layers, in a proper way. As a result, the C–C–T–T multi-stage layout was adapted for CoAtNet. where C and T denote Convolution and Transformer respectively. The CoAtNet architecture is shown in Fig. 3. Where L_i_ refers to number of blocks. The relative attention (rel-attenttion) is a result of unifying depthwise convolution and self-attention.

CoAtNet has several variants starting from CoAtNet-0 through CoAtNet-4. Conv layers, and MBConv blocks use kernel of size 3, while Transformer blocks use attention heads of size 32. As a result, 25 million of parameters have been trained in CoAtNet0.

Recently, CoAtNet has been used in numerous medical applications (e.g., medical image segmentation, image classification). Classification of skin cancer types using CoAtNet was demonstrated^60^. Additionally, brain tumor image segmentation was proposed utilizing CoAtNet^61^. Furthermore, CoAtNet was compared with EfficientNet-V2, and ResNext50 which are SOTA ConvNets and it was found to outperform them for bone marrow cells classification^62^.

## Experiments and results

This section describes the experiments conducted for parasitic eggs recognition in microscopic images. The setup of each experiment of model implementation is demonstrated. Additionally, the performance results such as accuracy, recall, precision, and f1 score are discussed. Furthermore, the comparison between CNN based, self-attention based, and CoAtNet based models is highlighted to show the superior performance of the proposed solution.

### Experimental setup

The experiments were conducted by training the CNN based models, self-attention vision transformer, and CoAtNet based model using a TensorFlow framework on a 1 or 2 NVIDIA Tesla V100 GPUs. The hyperparameters such as batch size, number of epochs, optimizer, and input size have been tuned several times to find the optimal values that can produce the best validation accuracy using the validation data. As seen, different hyperparameters were found for each model including EfficientNet, DenseNet, vision transformer, and CoAtNet.

The microscopic images were resized to 380 × 380 pixels for EfficientNet as mentioned in original paper^35^. In DenseNet, and CoAtNet, for image (input) size, 224 × 224 dimensions were used and applied to a network. After that, the 384 × 384 dimensions were applied. By comparing accuracy, it was found that increasing input size can improve classification accuracy remarkably.

The hyperparameters such as number of dense layers, and number of nodes in dense layer have been tuned several times to find the optimal values that can produce the best validation accuracy using the validation data. As seen, the optimal number of dense layers, and number of nodes in each are shown in Tables 3, 5, 7 and 9 for each model. As known, increasing number of dense layers and their nodes leads to increase in computation cost and may result in over fitting problem, and thus dropout layers were added to avoid this problem.

**Table 3:** Architecture of DenseNet121.

InputLayer (shape = (384,384,3))
DenseNet121(backbone)
GlobalAveragePooling2D ()
Dense (300)
ReLU activation function
Dropout (0.5)
Dense (100)
ReLU activation function
Dropout (0.5)
Dense (11)
Softmax activation function

Additionally, the images were normalized using the mean and standard deviation of ImageNet. The details of architectures and hyperparameters for each model including EfficientNet, DenseNet, vision transformer, and CoatNet are demonstrated in Tables 3, 4, 5, 6, 7, 8, 9 and 10. The labeled microscopic dataset includes 11,000 images. The dataset was divided into: training with 6600 images (60%), validation with 2200 images (20%), and testing with 2200 images (20%). Each of these subsets should have equal number of images belonging to eleven categories to avoid imbalance distribution.

**Table 4:** Hyperparameters of DenseNet121.

Mini_batch_size = 32
Number of GPU = 1
Epochs = 10
Optimizer = Adam

**Table 5:** Architecture of EfficentNet-B4.

InputLayer (shape = (380,380,3))
EfficientNet-B4(backbone)
GlobalAveragePooling2D ()
BatchNormalization
Dropout (0.5)
Dense (1024)
ReLU activation function
Dense (512)
ReLU activation function
BatchNormalization
Dropout (0.5)
Dense (11)
Softmax activation function

**Table 6:** Hyperparameters of EfficentNet-B4.

Mini_batch_size = 64
Number of GPU = 1
Epochs = 20
Optimizer = Adam

**Table 7:** Architecture of vision transformer.

InputLayer (shape = (224,224,3))
ViT-B_16 (backbone)
Dense (512)
ReLU
Dense (11)
Softmax activation function

**Table 8:** Hyperparameters of vision transformer.

Mini_batch_size = 16
Gpu_num = 1
Epochs = 8
Optimizer = Adam

**Table 9:** Architecture of CoAtNet0.

InputLayer (shape = (384,384,3))
CoatNet0(backbone)
GlobalAveragePooling2D ()
Dense (11)
Softmax activation function

**Table 10:** Hyperparameters of CoAtNet0.

Mini_batch_size_per_gpu = 8
Gpu_num = 2
Epochs = 8
Optimizer = Adam
Reduce_on_plateau_settings = {factor = 0.1,min_lr = 0.00001,patience = 2}

### Results discussion

This section discusses the results of three experiments carried out to classify parasitic eggs in microscopic images into 11 classes. The performance metrics including accuracy, recall, precision, and F1 scores were calculated in each experiment. The comparison between DenseNet121, EfficientNet-B4, vision transformer (vit-16 base), and CoAtNet0 was done to evaluate and highlight the capability of each method in parasitic egg recognition task.

The first experiment was done to evaluate the performance of EfficientNet-B4 CNN. The version B4 was selected because it can balance between high accuracy and high speed of training and inference. Various numbers of layers tuned were evaluated to find the best hyperparameters that were able to produce the highest performance in terms of accuracy, recall, precision, and F1 scores. Tables 11, 12, and 13 show the classification metrics for each category in each of three scenarios: tuning last 5 layers, tuning last 20 layers, and tuning last 40 layers, respectively.

**Table 11:** Classification report of the EfficientNet-B4 after tuning last 5 layers.

	Precision	Recall	f1-score
*Ascaris lumbricoides*	0.63	0.46	0.53
*Capillaria philippinensis*	0.62	0.86	0.73
*Enterobius vermicularis*	0.77	0.87	0.82
*Fasciolopsis buski*	0.89	0.69	0.78
*Hookworm egg*	0.94	0.97	0.96
*Hymenolepis diminuta*	0.69	0.79	0.73
*Hymenolepis nana*	0.85	0.80	0.82
*Opisthorchis viverrine*	0.81	0.64	0.71
*Paragonimus* spp.	0.83	0.59	0.69
*Taenia* spp. egg	0.69	0.78	0.73
*Trichuris trichiura*	0.64	0.81	0.71

**Table 12:** Classification report of the EfficientNet-B4 after tuning last 20 layers.

	Precision	Recall	f1-score
*Ascaris lumbricoides*	0.84	0.68	0.75
*Capillaria philippinensis*	0.54	0.98	0.69
*Enterobius vermicularis*	0.83	0.96	0.89
*Fasciolopsis buski*	0.93	0.84	0.88
*Hookworm egg*	0.96	0.98	0.97
*Hymenolepis diminuta*	0.66	0.79	0.72
*Hymenolepis nana*	0.94	0.81	0.87
*Opisthorchis viverrine*	0.95	0.62	0.75
*Paragonimus* spp.	0.81	0.65	0.72
*Taenia* spp. egg	0.94	0.74	0.83
*Trichuris trichiura*	0.85	0.82	0.84

**Table 13:** Classification report of the EfficientNet-B4 after tuning last 40 layers.

	Precision	Recall	f1-score
*Ascaris lumbricoides*	0.90	0.78	0.83
*Capillaria philippinensis*	0.87	0.89	0.88
*Enterobius vermicularis*	0.92	0.98	0.95
*Fasciolopsis buski*	0.95	0.95	0.95
*Hookworm egg*	0.99	0.99	0.99
*Hymenolepis diminuta*	0.74	0.93	0.82
*Hymenolepis nana*	0.97	0.94	0.96
*Opisthorchis viverrine*	0.90	0.84	0.87
*Paragonimus* spp.	0.93	0.69	0.80
*Taenia* spp. egg	0.91	0.96	0.93
*Trichuris trichiura*	0.90	0.95	0.93

**Figure 4:**
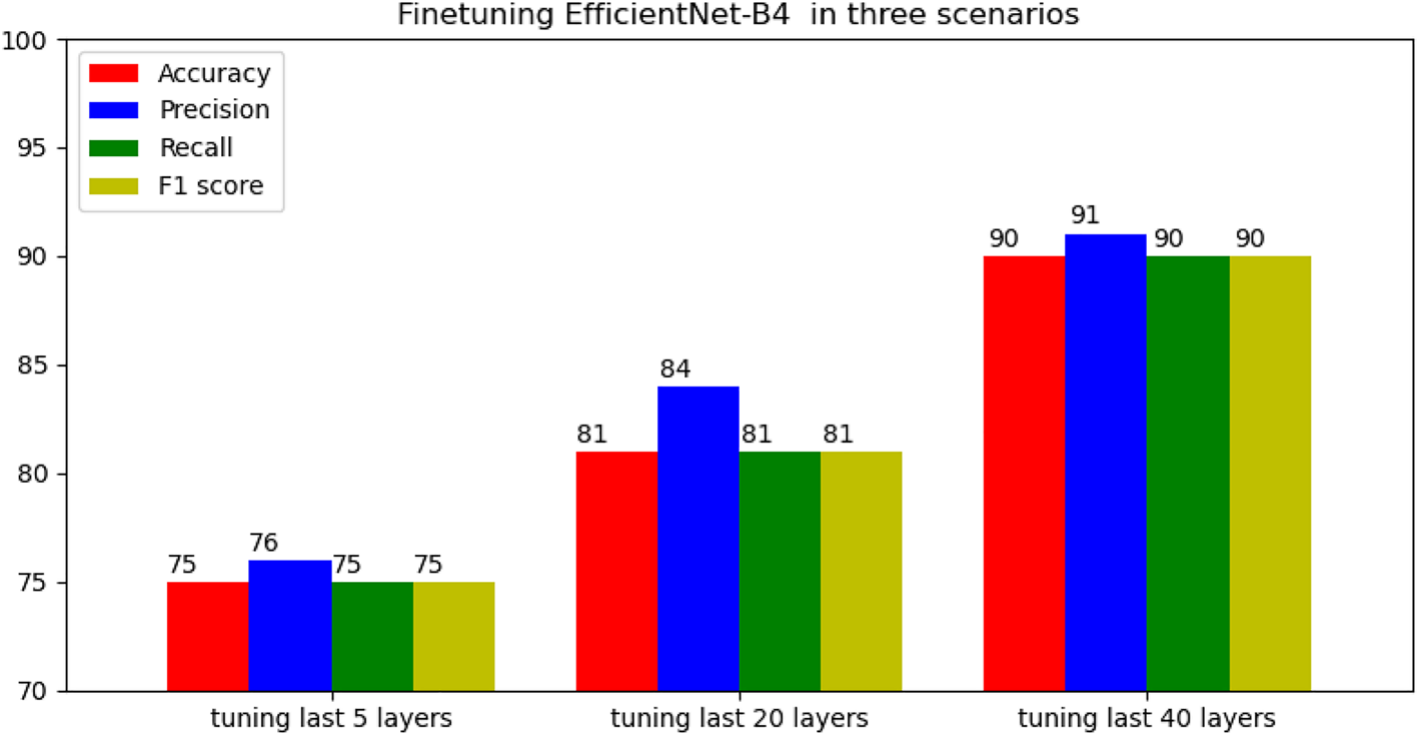
Finetuning EfficientNet-B4 in three scenarios.

The recall, precision, and F1 scores were calculated for each class out of eleven classes as shown in Fig. 4. Then, the average of recall, precision, and F1 scores were found. Additionally, the average of accuracy was found. It is obvious that tuning last 40 layers of EfficientNet-B4 CNN was able to give the highest results in terms of average accuracy (90%), average recall (90%), average precision (91%), and average F1 score (90%). On the other hand, tuning only last 5 layers was not efficient and the metrics produced were low with average accuracy (75%), average recall (75%), average precision (76%), and average F1 score (75%). The EfficientNet was pre-trained with ImageNet dataset which includes 1000 classes that are different from parasitic egg classes. The low metric of tuning only last 5 layers indicated that parameters of EfficientNet-B4 CNN were not able to extract informative features or presentations that were suitable for parasitic egg recognition. In other words, there was a need to tune more layers (last 40 layers scenario) to learn better parameters and more informative features that can differentiate between various types or categories of parasitic eggs. Additionally, the results of tuning last 40 layers show high F1 scores of recognizing Hookworm egg and Hymenolepis nana types of eggs. On the other hand, the results show low F1 scores for three classes of *Paragonimus* spp, *Hymenolepis diminuta*, and *Ascaris lumbricoides* which indicated inability of EfficientNet-B4 to distinguish between these types of eggs.

**Figure 5:**
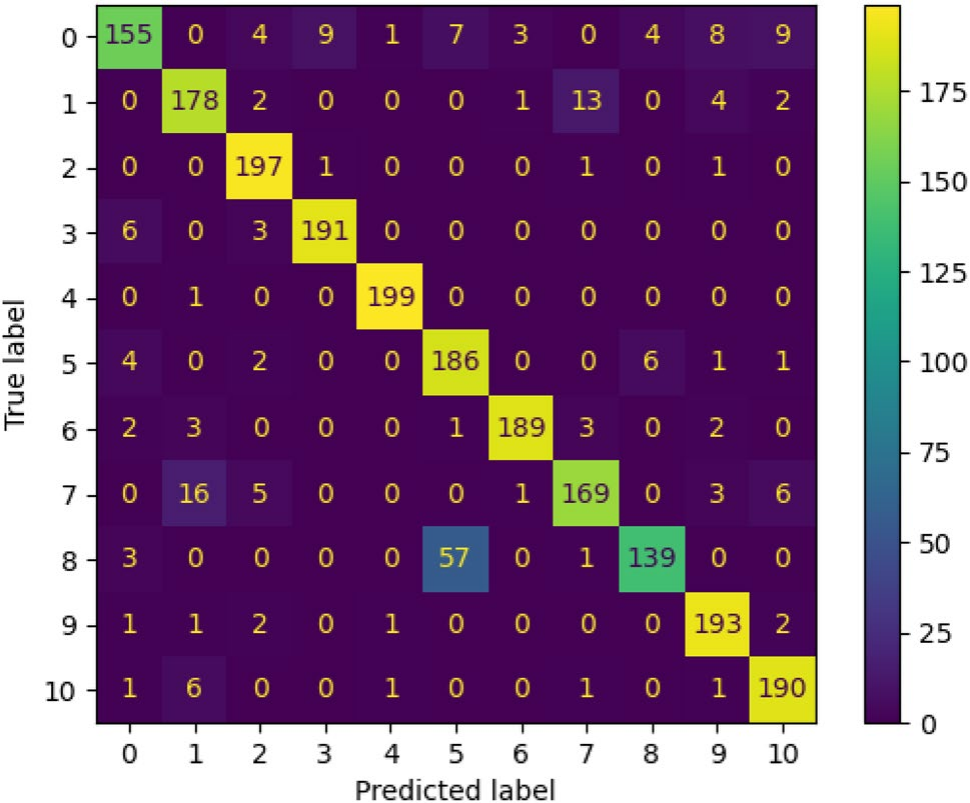
Confusion matrix utilizing EfficientNetB4 after tuning 40 layers.

The confusion matrix of EfficientNet-B4 CNN after tuning the last 40 layers is shown in Fig. 5. The high values of elements in the main diagonal refer to a high accuracy of the model to recognize the parasitic eggs. The class 4 (Hookworm egg) was recognized perfectly with 199/200 correct predictions. On the other hand, the class 8 (*Paragonimus* spp.) was misclassified largely compared to other classes with 139/200 correct predictions. The majority of misclassified samples in class 8 were classified wrongly as class 5 (*Hymenolepis diminuta*) which indicated the similarity between features extracted by EfficientNet-B4 from microscopic images that belong to classes 5 and 8.

**Figure 6:**
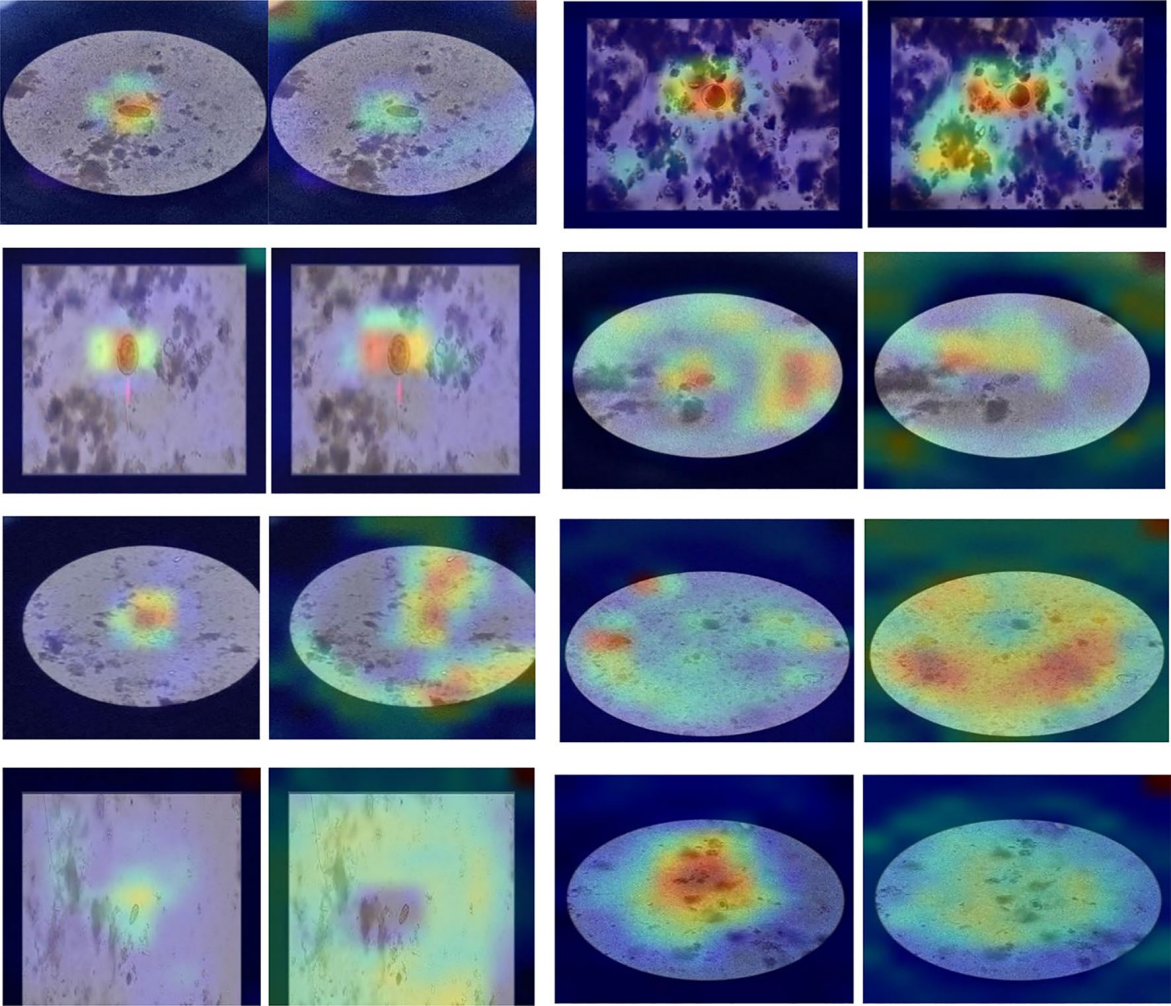
Activation maps of various classes utilizing EfficientNetB4, where the left images are the activation maps after tuning the network, and the right images are the activation maps after freezing the network.

Figure 6 shows activation maps of various parasitic egg classes utilizing EfficientNet-B4 before tunning (freezing layers) and after tuning of 40 layers. The maps illustrate the capability of EfficientNet being tuned to focus the attention on the objects (eggs) inside the microscopic images and ignore irrelevant staff. On the contrary, EficientNet-B4 with layers frozen was unable to highlight regions in the image that were relevant to the class of egg.

The second experiment was done to evaluate the performance of DenseNet CNN. Different versions of DenseNet such as 121, 169, and 201 were evaluated and compared. The DenseNet with 121 layers was selected because it can balance between high accuracy and high speed of training and inference. Various numbers of layers tuned were evaluated to find the best hyperparameters that were able to produce the highest performance in terms of accuracy, recall, precision, and F1 scores. Table 14 shows the classification report for scenario of tuning last 20 layers. The recall, precision, and F1 scores were calculated for each class from eleven classes. Then, the average of recall, precision, and F1 scores were found. Additionally, the average of accuracy was found. It is obvious that tuning last 20 layers of DenseNet121 CNN was able to give high results in terms of average accuracy (86%), average recall (86%), average precision (88%), and average F1 score (86%). The DenseNet121 was pre-trained with ImageNet dataset which includes 1000 classes that are different from parasitic egg classes. The scenario of tuning last 20 layers was found to be able to extract informative features that were suitable for parasitic egg recognition. Additionally, it learnt better parameters that can differentiate between various types or categories of parasitic eggs. The results of tuning last 20 layers showed high F1 scores for Hookworm egg and Fasciolopsis buski types of eggs. On the other hand, the results showed low F1 score of class Capillaria philippinensis which indicated inability of DenseNet121 to distinguish this type of parasitic egg.

**Table 14:** Classification report of the DenseNet121 after tuning last 20 layers.

	Precision	Recall	F1-score	Support
*Ascaris lumbricoides*	0.90	0.78	0.83	200
*Capillaria philippinensis*	0.75	0.84	0.79	200
*Enterobius vermicularis*	0.80	0.98	0.88	200
*Fasciolopsis buski*	0.99	0.89	0.93	200
*Hookworm egg*	0.99	0.97	0.98	200
*Hymenolepis diminuta*	0.72	0.96	0.83	200
*Hymenolepis nana*	1.00	0.80	0.89	200
*Opisthorchis viverrine*	0.80	0.88	0.84	200
*Paragonimus* spp	0.97	0.72	0.83	200
*Taenia* spp. egg	0.81	0.96	0.88	200
*Trichuris trichiura*	0.97	0.72	0.83	200
Accuracy			**0.86**	2200
Macro avg	**0.88**	**0.86**	**0.86**	2200

Significant values are in [bold].

**Figure 7:**
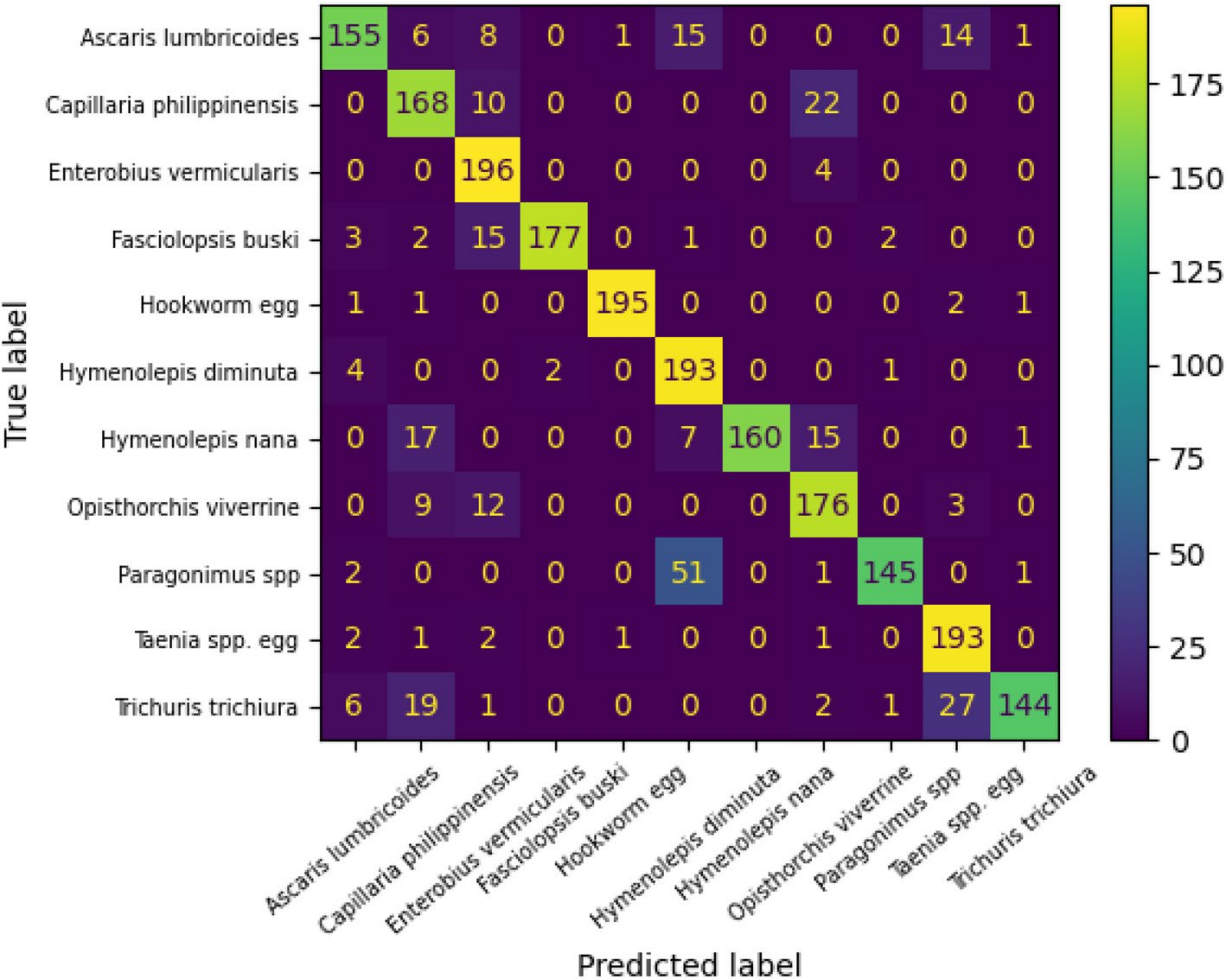
Confusion matrix of the DenseNet121 after tuning 20 layers.

The confusion matrix of DenseNet121 CNN after tuning the last 20 layers is shown in Fig. 7. The high values of elements in the main diagonal refer to high accuracy of the model to recognize the parasitic eggs. The classes 2 and 4 (Enterobius vermicularis and Hookworm egg) were recognized perfectly with 196/200 and 195/200, respectively of correct predictions. On the other hand, the class 8 (P*aragonimus* spp.) was misclassified largely compared to other classes with 145/200 correct predictions. The majority of misclassified samples in class 8 were classified wrongly as class 5 (*Hymenolepis diminuta*) which indicated the similarity between features extracted by DenseNet121 from microscopic images that belong to classes 5 and 8.

**Figure 8:**
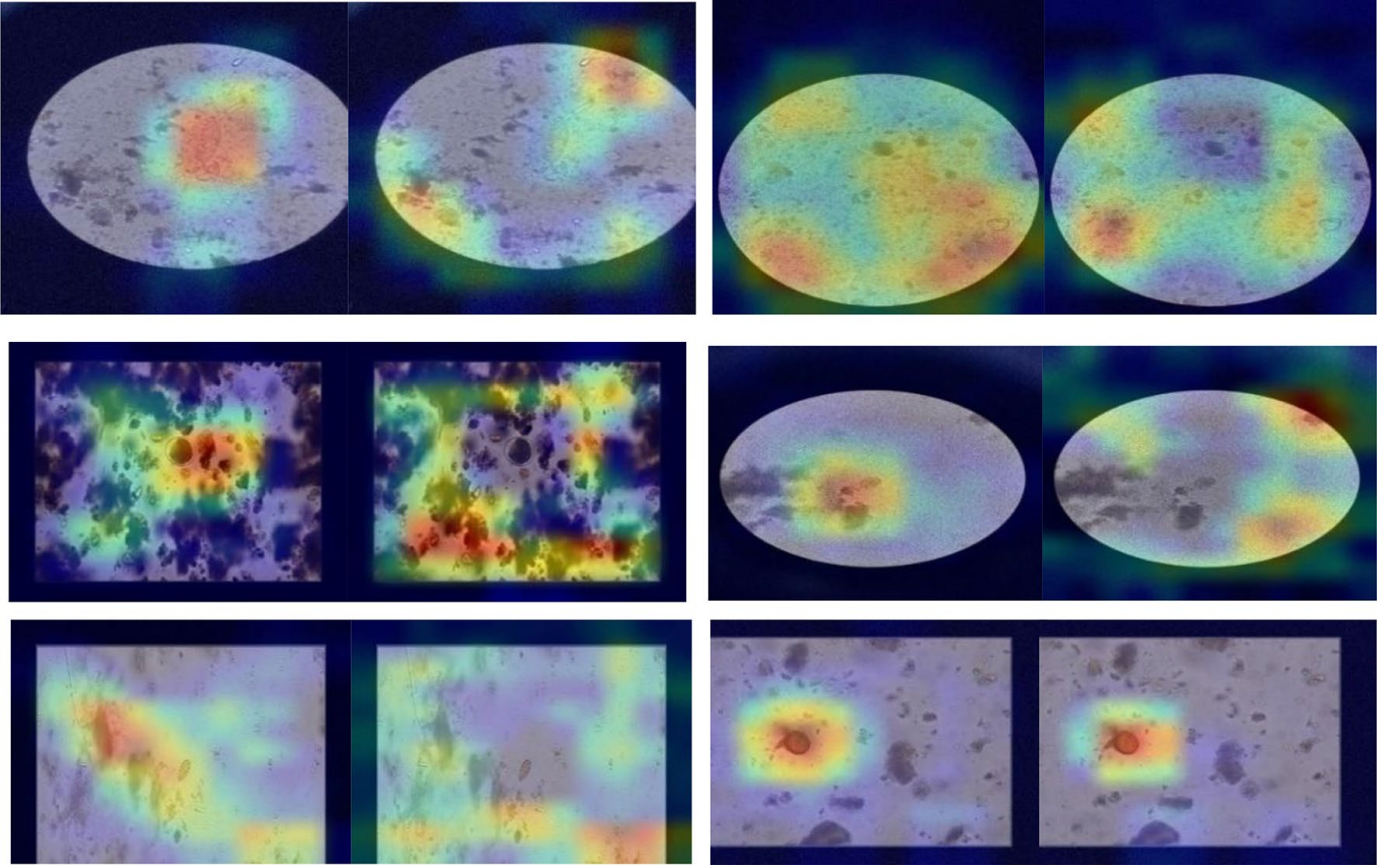
Activation maps of various classes utilizing DenseNet121, where the left images are the activation maps after tuning the network, and the right images are the activation maps after freezing the network.

Figure 8 shows activation maps of various parasitic egg classes utilizing DenseNet121 before tunning (freezing layers) and after tuning of 20 layers. The maps illustrate the capability of DenseNet being tuned to focus the attention on the objects (eggs) inside the microscopic images and ignore irrelevant staff. On the contrary, Densenet121 with layers frozen was unable to highlight regions in the image that were relevant to the class of egg.

The third experiment was done to evaluate the performance of vision transformer. The version 16 was selected because it has fewer parameters. The vision transformer was pre-trained on ImageNet-21k, a dataset consisting of 14 million images and 21 k classes, and fine-tuned on ImageNet, a dataset consisting of 1 million images and 1 k classes. These datasets are different from parasitic egg classes. In Table 15, the recall, precision, and F1 scores were calculated for each class from eleven classes.

**Table 15:** Classification report of the vision transformer after tuning all layers.

	Precision	Recall	F1-score	Support
*Ascaris lumbricoides*	0.93	0.92	0.92	200
*Capillaria philippinensis*	0.63	0.97	0.77	200
*Enterobius vermicularis*	0.67	0.99	0.80	200
*Fasciolopsis buski*	0.99	0.94	0.97	200
*Hookworm egg*	0.94	0.99	0.97	200
*Hymenolepis diminuta*	0.84	0.86	0.85	200
*Hymenolepis nana*	1.00	0.82	0.90	200
*Opisthorchis viverrine*	0.99	0.41	0.58	200
*Paragonimus* spp	0.87	0.86	0.86	200
*Taenia* spp. egg	0.99	0.81	0.89	200
*Trichuris trichiura*	0.99	0.89	0.93	200
Accuracy			**0.86**	2200
Macro avg	**0.89**	**0.86**	**0.86**	2200

Significant values are in [bold].

The fourth experiment was done to evaluate the performance of CoAtNet. The version 0 was selected because it has fewer parameters, fewer blocks and channels and can produce higher accuracy than other CNN-only based models. The CoAtNet was pre-trained with ImageNet dataset which includes 1000 classes that are different from parasitic egg classes. In Tables 16 and 17, the recall, precision, and F1 scores were calculated for each class from eleven classes.

**Table 16:** Classification report of the CoAtNet0 after tuning only the classification head.

	Precision	Recall	F1-score
*Ascaris lumbricoides*	0.61	0.34	0.44
*Capillaria philippinensis*	0.56	0.82	0.67
*Enterobius vermicularis*	0.73	0.81	0.77
*Fasciolopsis buski*	0.69	0.87	0.77
*Hookworm egg*	0.95	0.95	0.95
*Hymenolepis diminuta*	0.55	0.64	0.59
*Hymenolepis nana*	0.96	0.75	0.84
*Opisthorchis viverrine*	0.65	0.65	0.65
*Paragonimus* spp.	0.66	0.42	0.51
*Taenia* spp. egg	0.88	0.79	0.83
*Trichuris trichiura*	0.69	0.81	0.75

**Table 17:** Classification report of the CoAtNet0 after tuning all layers.

	Precision	Recall	F1-score
*Ascaris lumbricoides*	0.81	0.99	0.89
*Capillaria philippinensis*	0.92	0.97	0.95
*Enterobius vermicularis*	0.92	0.98	0.95
*Fasciolopsis buski*	0.93	0.87	0.90
*Hookworm egg*	1.00	1.00	1.00
*Hymenolepis diminuta*	0.97	0.70	0.82
*Hymenolepis nana*	0.99	0.98	0.98
*Opisthorchis viverrine*	0.99	0.89	0.94
*Paragonimus* spp.	0.82	0.90	0.86
*Taenia* spp. egg	1.00	0.97	0.99
*Trichuris trichiura*	0.97	0.99	0.98

**Figure 9:**
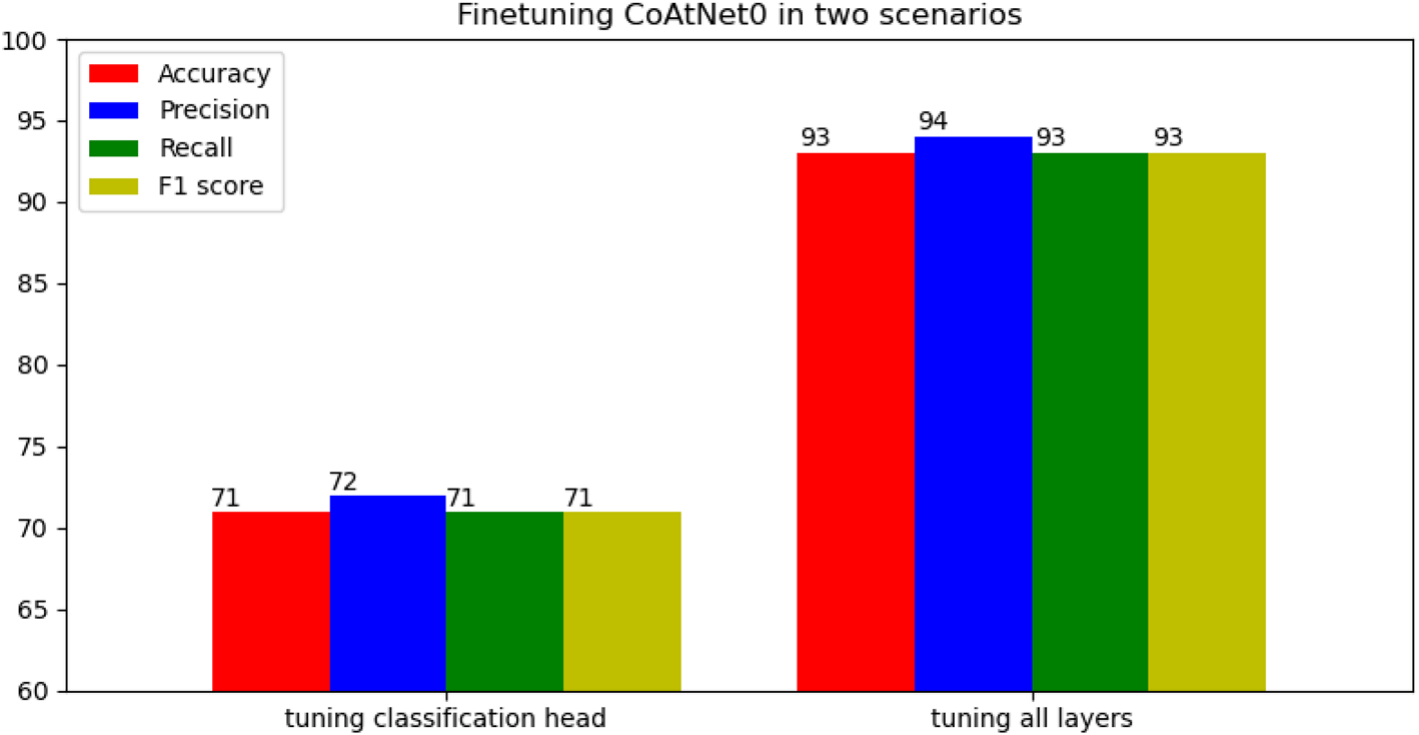
Finetuning CoAtNet0 in two scenarios.

Then, the average of recall, precision, and F1 scores were found. Additionally, the average of accuracy was found. There were two scenarios to use CoAtNet as shown Fig. 9. The first scenario was to freeze all layers in the backbone and tune only head classification layers. This scenario produced the worst performance in terms of accuracy, recall, precision, and F1 scores as shown in Fig. 9 with 71% average accuracy, and 71% average F1 score. Freezing all layers of backbone indicated that parameters of CoAtNet CNN were not able to extract informative features or presentations that were suitable for parasitic egg recognition. In other words, there was a need to tune more layers to learn better parameters and more informative features that can differentiate between various types or categories of parasitic eggs. Therefore, the second scenario was implemented to tune all layers in the CoAtNet backbone. This scenario was able to give the highest performance in terms of average accuracy (93%), average recall (93%), average precision (94%), and average F1 score (93%) as shown in Fig. 9. The results of tuning all layers showed high F1 scores for the most of parasitic egg types. On the other hand, the results showed low F1 scores for two classes of *Hymenolepis diminuta* (82%), and *Paragonimus* spp (86%). However, the F1 score of *Paragonimus* spp in CoAtNet0 is still higher than one of EfficientNet-B4 (80%) and one of DenseNet121 (83%).

**Figure 10:**
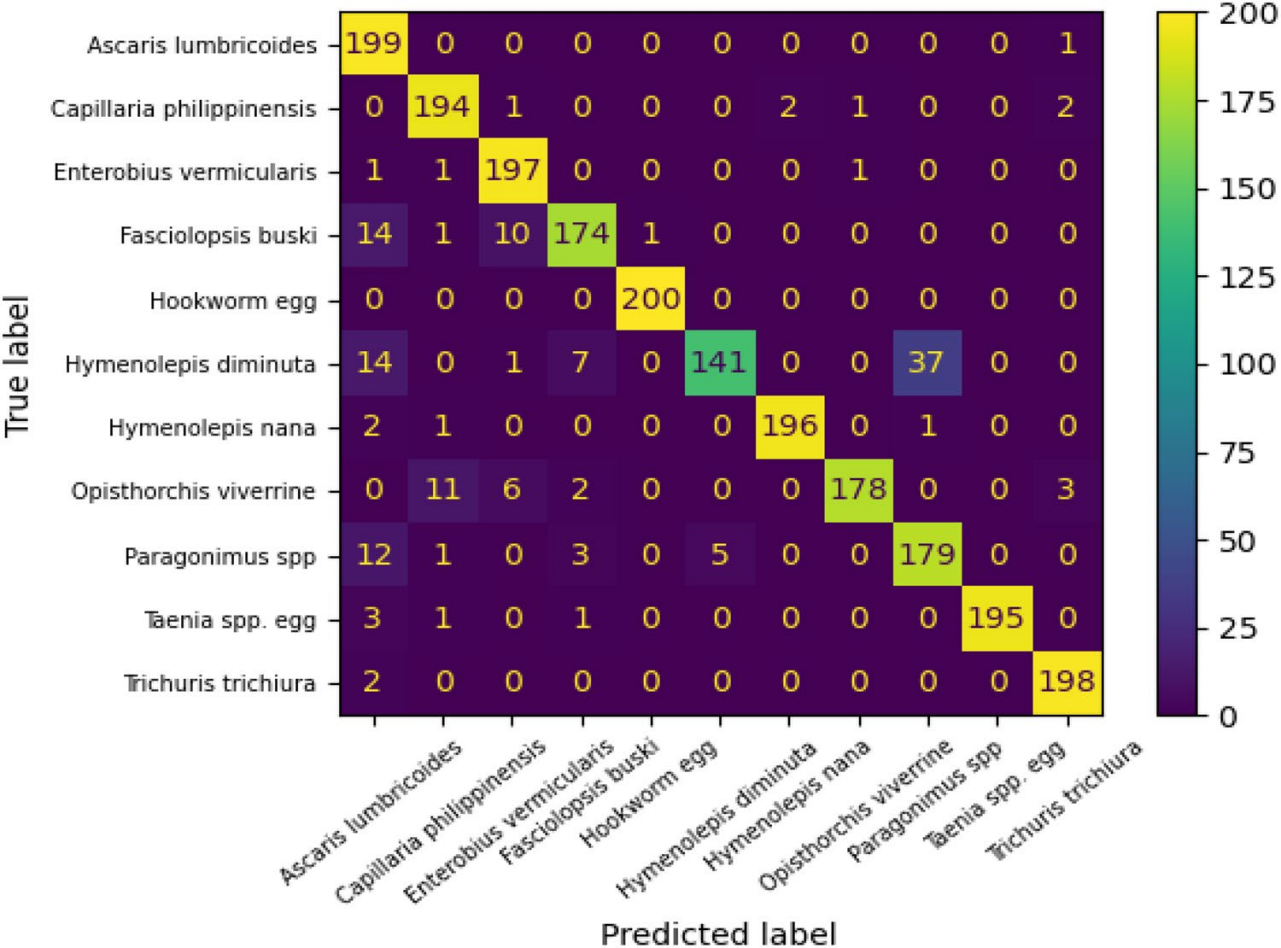
Confusion matrix of the CoatNet0 after tuning all layers.

The confusion matrix of CoAtNet after tuning all layers is shown in Fig. 10. The high values of elements in the main diagonal refer to high accuracy of the CoAtNet to recognize the parasitic eggs. The classes 0, 1, 2, 4, 6, 9, and 10 were recognized perfectly with 199, 194, 197, 200, 196, 195, and 198 of correct predictions out of 200, respectively. On the other hand, the class 5 (*Hymenolepis diminuta*) was misclassified largely compared to other classes with 141/200 correct predictions. The majority of misclassified samples in class 5 were classified wrongly as class 8 (*Paragonimus* spp.) which indicated the similarity between features extracted by CoAtNet from microscopic images that belong to classes 5 and 8. Additionally, the classes 0 and 1 of Ascaris lumbricoides and Capillaria philippinensis were recognized well in CoAtNet (199/200 and 194/200 respectively) compared to (155/200 and 178/200 respectively in EfiientNet-B4) and (155/200 and 168/200 respectively in DenseNet121).

**Figure 11:**
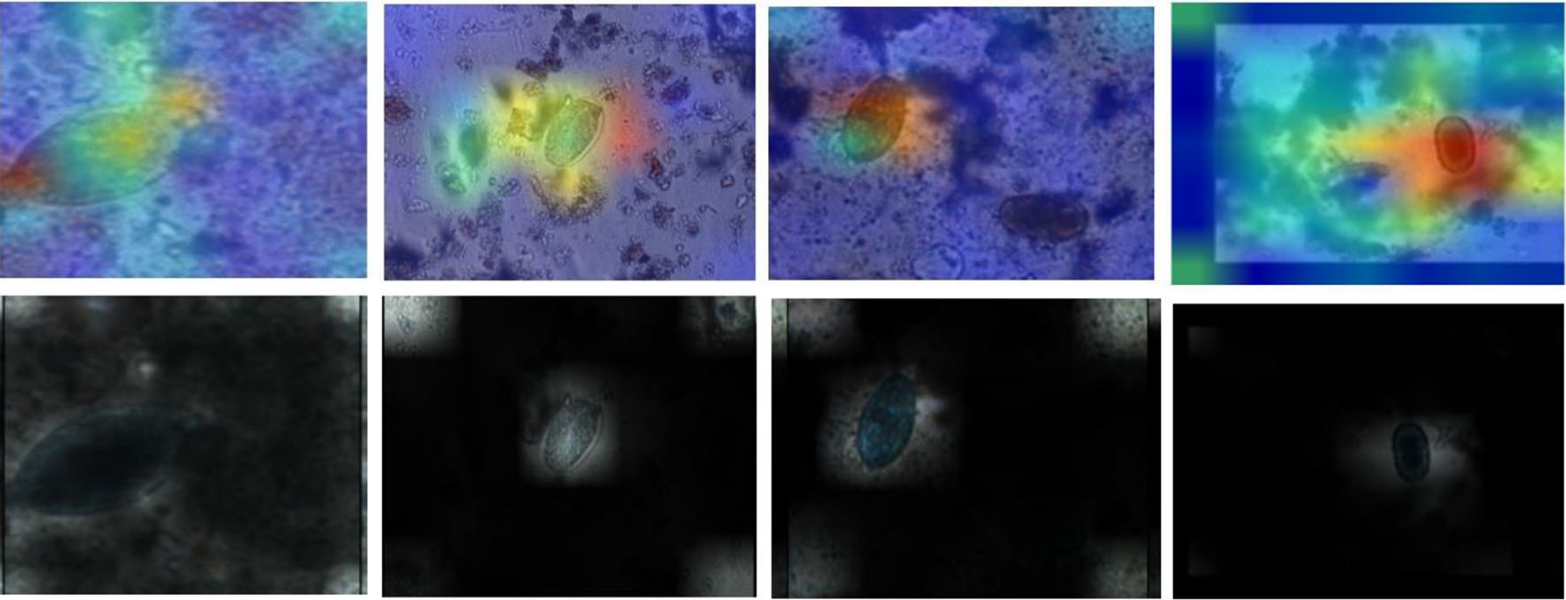
Activation maps of various classes of CoAtNet0 (first row) and attention score visualization of CoAtNet0 (second row).

Figure 11 shows activation maps and attention score of various parasitic egg classes utilizing CoAtNet0. The maps and scores illustrate the capability of CoAtNet0 to focus the attention on the objects (eggs) inside the microscopic images and ignore irrelevant staff.

**Figure 12:**
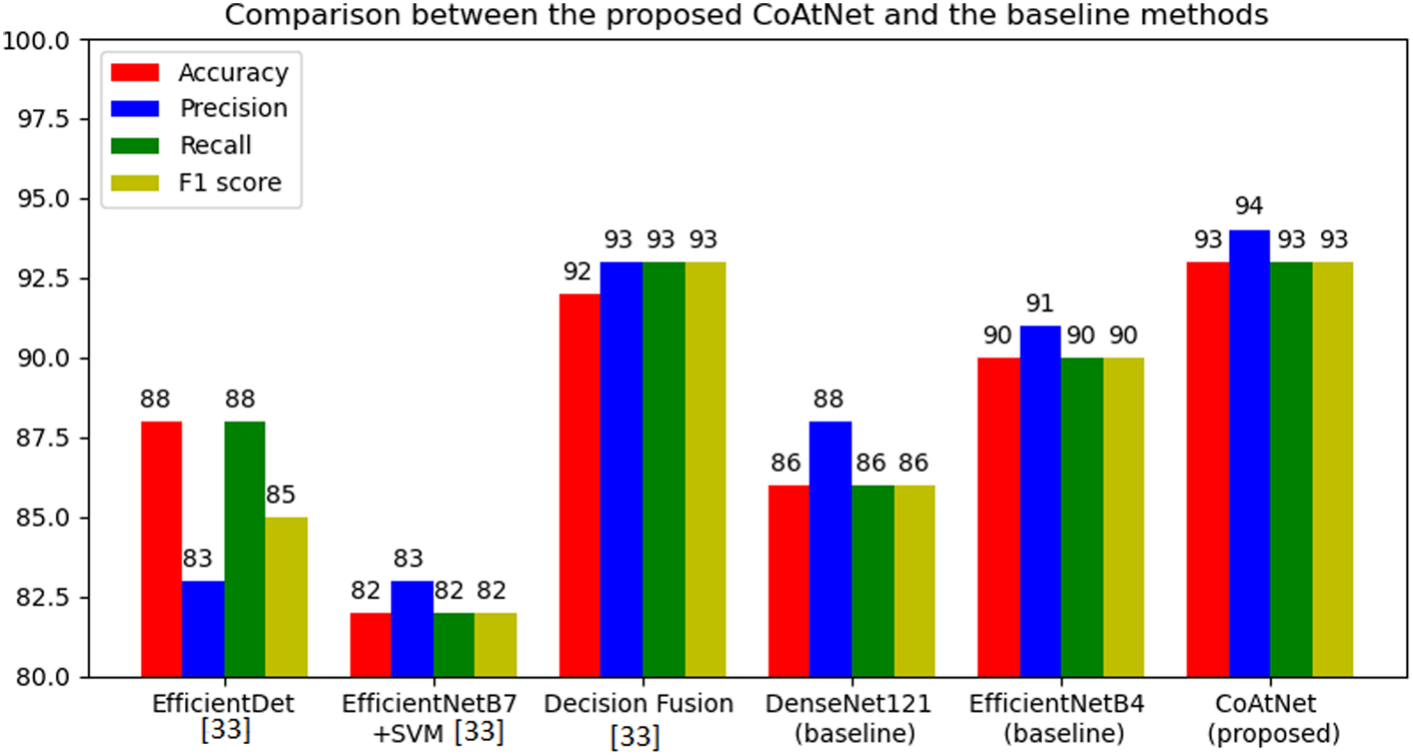
Comparison between the proposed CoAtNet and the baseline methods.

Figure 12 compares between the proposed solution of CoAtNet and several baselines including CNN-only based models such as EfficientNet-B4 with 40 layers tuned (90% accuracy and 90% F1 score), DenseNet121with 20 layers tuned (86% accuracy and 86% F1 score), and EfficientNet-B7 with layers frozen and support vector machine (SVM) tuned^33^ (82% accuracy and 82% F1 score). Similarly, Fig. 12 compares CoAtNet with other methods used for parasitic egg recognition^33^ such as EfficientDet with EfficientNet-v2 backbone^33^ (88% accuracy and 85% F1 score). The results showed superior performance of convolution and attention network method (93% accuracy and 93% F1 score) compared to baseline methods. Additionally, the CoAtNet0 that has lower computational cost was found to outperform the fusion decision method (92% accuracy and 93% F1 score)^33^ that has complex architecture of two backbones used to extract features and fuse the decision produced in the output layers.

The combination of two techniques including attention and convolution in CoAtNet plays role to improve parasitic egg recognition because it combines properties of both models (convolutional network and vision transformer). The convolutional neural network gives the task of parasitic egg recognition more generalization, and efficiency. On the other hand, the vision transformer gives the task larger model capacity that can benefits from the large parasitic egg dataset. As a result, the previous advantages of this combination lead to produce a higher testing accuracy that cannot be achieved by a standalone convolutional network such as EfficientNet and DenseNet.

Finally, it is necessary to study the cost of each model in terms of time complexity and memory efficiency. As shown in Table 18, CoAtNet0 was able to balance between inference time and number of parameters compared to CNN bae models (DenseNet121 and EfficientNet-B4) and self-attention-based vision transformer. CoAtNet0 can produce lower inference time and number of parameters than vision transformer. However, CoAtNet0 has higher inference time and number of parameters than EfficiientNet-B4 and DenseNet121.

**Table 18:** Comparison between methods in terms of inference time and memory efficiency.

Architecture	Number of parameters	Inference time (s)	Flops (B)
DenseNet121	7,376,215	0.082	16.8
EfficientNet-B4	20,049,514	0.094	9
ViT-B_16	86,198,027	0.111	35.2
**CoatNet0 (proposed)**	**22,623,557**	**0.095**	**26.3**

This paper shaded light on an interesting challenge and contributed to the body of knowledge as follows:

1. Various pre-trained CNNs such as DenseNet121 and EfficientNet-B4, and pre-trained vision transformer were fine-tuned with parasitic eggs dataset for classification purposes. EfficientNet-B4 that has five layers tuned got the worst performance (75% accuracy and 75% F1 score). On the other hand, EfficientNet-B4 that has 40 layers tuned produced the best performance (90% accuracy and 90% F1 score). Additionally, DenseNet121 that has 20 layers tuned gave 86% accuracy and 86% F1 score. Furthermore, vision transformer gave 86% accuracy and 86% F1 score.
2. A novel solution utilizing the concept of combining convolution and attention to recognize eleven types of parasitic eggs. A CoAtNet was proposed for parasitic egg recognition in microscopic images for classifying eggs into eleven categories. The pre-trained CoAtNet that was used after freezing all layers except the classification heads gave the worst performance of 71% accuracy and 71% F1 score. On the other hand, pre-trained CoAtNet with all layers tuned produced the best performance of 93% accuracy and 93% F1 score.
3. This work targeted a novel dataset that was proposed in ICIP2022 challenge to recognize eleven types of parasitic eggs acquired under various complex conditions such as illuminations and resolutions. The proposed solution of convolution and attention was able to outperform not CNN-only based models and self-attention vision transformer, but also object detection method of EfficientDet with EfficientNet-v2 backbone. This finding proposes a novel technique of adding CoAtNet as a backbone in object detector to enhance the detection performance.

## Conclusion and future work

This paper presented a fast, and highly accurate technique to recognize parasitic eggs in microscopic images and classify them into eleven categories. Various methods such as convolutional neural network (CNN) based models and convolution and attention (CoAtNet) based models were evaluated and compared using Chula-ParasiteEgg microscopic image dataset that was proposed in ICIP2022. The proposed identification technique of CoAtNet was trained and fine-tuned with eleven types of parasitic eggs including various egg sizes and numerous conditions such as illumination, resolution, and blurring. A CoAtNet was found to reduce diagnosis errors and give high sensitivity. It was able to outperform other CNN based models such as EfficientNet-b4 and DenseNet121 and self-attention based models such as vision transformer. An average accuracy of 93%, and an average F1 score of 93% were resulted from the proposed solution which helps to diagnose the most common intestinal parasitic infections in humans in low-and-middle-income countries. This also contributed to preserve the status of economic and health sectors in countries. The finding opens door to integrate the proposed solution in automated parasitological diagnosis.

In this study, the need to tune all layers of model requires powerful machine with multiple GPUs and large size of RAM memory to fine-tune large number of parameters which is costly process. Therefore, we used CoAtNet0 version because it has lower number of parameters compared to other versions to reduce the training time and hardware requirement and thus reduce the cost. Additionally, the selection of the proposed CoAtNet0 has a limitation related to inability to localize and classify parasitic eggs if more than one egg with different categories are available in the same microscopic image.

Hence, we intend to enhance the recognition performance by training bigger versions of convolution and attention networks, but this improvement requires more resources such as cloud platform with cluster of several machines, several GPUs, and memory. Additionally, the results showed superior performance of convolution and attention network compared to convolution-only models and thus they highlighted the potential of adding CoAtNet as a backbone in object detector to detect and classify multiple eggs with different categories in one microscopic image. Furthermore, augmentation of microscopic images by blurring and adding noise can enhance the recognition performance.

## Data Availability

The data that support the findings of this study are available from University of Bristol, UK, and Chulalongkorn University, Thailand under license Creative Commons Attribution. The data are publicly available in this link: https://ieee-dataport.org/competitions/parasitic-egg-detection-and-classification-microscopic-images.
