# Author Correction: Parasitic egg recognition using convolution and attention network

**DOI:** 10.1038/s41598-023-43068-z

**Published:** 2023-09-22

**Authors:** Nouar AlDahoul, Hezerul Abdul Karim, Mhd Adel Momo, Francesca Isabelle F. Escobar, Vina Alyzza Magallanes, Myles Joshua Toledo Tan

**Affiliations:** 1 Computer Science, New York University, Abu Dhabi, United Arab Emirates; 2 Faculty of Engineering, Multimedia University, Cyberjaya, Malaysia; 3 Fleet Management Systems and Technologies, Istanbul, Turkey; 4 Department of Natural Sciences, University of St. La Salle, Bacolod, Philippines

Correction to: *Scientific Reports* 10.1038/s41598-023-41711-3, published online 02 September 2023

In the original version of this Article Hezerul Abdul Karim was omitted as a corresponding author. Correspondence and requests for materials should also be addressed to hezerul@mmu.edu.my.

The original Article has been corrected.

